# Triceps Surae Muscle-Tendon Properties as Determinants of the Metabolic Cost in Trained Long-Distance Runners

**DOI:** 10.3389/fphys.2021.767445

**Published:** 2022-01-04

**Authors:** Esthevan Machado, Fábio Juner Lanferdini, Edson Soares da Silva, Jeam Marcel Geremia, Francesca Chaida Sonda, Jared R. Fletcher, Marco Aurélio Vaz, Leonardo Alexandre Peyré-Tartaruga

**Affiliations:** ^1^Laboratório de Pesquisa do Exercício, Universidade Federal do Rio Grande do Sul, Porto Alegre, Brazil; ^2^Department of Health and Physical Education, Mount Royal University, Calgary, AB, Canada; ^3^Laboratório de Biomecânica, Universidade Federal de Santa Catarina, Florianópolis, Brazil

**Keywords:** long-distance runners, calf muscles, muscle architecture, Achilles tendon properties, metabolic cost

## Abstract

**Purpose:** This study aimed to determine whether triceps surae’s muscle architecture and Achilles tendon parameters are related to running metabolic cost (C) in trained long-distance runners.

**Methods:** Seventeen trained male recreational long-distance runners (mean age = 34 years) participated in this study. C was measured during submaximal steady-state running (5 min) at 12 and 16 km h^–1^ on a treadmill. Ultrasound was used to determine the gastrocnemius medialis (GM), gastrocnemius lateralis (GL), and soleus (SO) muscle architecture, including fascicle length (FL) and pennation angle (PA), and the Achilles tendon cross-sectional area (CSA), resting length and elongation as a function of plantar flexion torque during maximal voluntary plantar flexion. Achilles tendon mechanical (force, elongation, and stiffness) and material (stress, strain, and Young’s modulus) properties were determined. Stepwise multiple linear regressions were used to determine the relationship between independent variables (tendon resting length, CSA, force, elongation, stiffness, stress, strain, Young’s modulus, and FL and PA of triceps surae muscles) and C (J kg^–1^m^–1^) at 12 and 16 km h^–1^.

**Results:** SO PA and Achilles tendon CSA were negatively associated with C (*r*^2^ = 0.69; *p* < 0.001) at 12 km h^–1^, whereas SO PA was negatively and Achilles tendon stress was positively associated with C (*r*^2^ = 0.63; *p* = 0.001) at 16 km h^–1^, respectively. Our results presented a small power, and the multiple linear regression’s cause-effect relation was limited due to the low sample size.

**Conclusion:** For a given muscle length, greater SO PA, probably related to short muscle fibers and to a large physiological cross-sectional area, may be beneficial to C. Larger Achilles tendon CSA may determine a better force distribution per tendon area, thereby reducing tendon stress and C at submaximal speeds (12 and 16 km h^–1^). Furthermore, Achilles tendon morphological and mechanical properties (CSA, stress, and Young’s modulus) and triceps surae muscle architecture (GM PA, GM FL, SO PA, and SO FL) presented large correlations with C.

## Introduction

Economical running is an important biological advantage in human evolution (Saibene and Minetti, 2003). During the evolutionary process, adaptations at the organismal level (e.g., lower limbs morphology) allowed humans to run greater distances with a lower energy cost compared to their primate relatives (Steudel-Numbers and Wall-Scheffler, 2009). In this regard, human running has been classified as an energy-saving mechanism, where part of the total mechanical energy associated with the body center of mass is stored as elastic strain energy by the lower limb elastic structures (Cavagna et al., 1964; Alexander, 1991; Saibene and Minetti, 2003).

Endurance running performance is significantly related to some physiological factors, such as maximal oxygen uptake (V̇O_2MAX_), physiological transition thresholds, V̇O_2MAX_ fractional utilization, and the net energy cost of running [the energy expended above the resting energy expenditure per unit of distance, running metabolic cost (C), measured in J kg^–1^km^–1^] (di Prampero et al., 1986; Joyner and Coyle, 2008; McLaughlin et al., 2010; Kipp et al., 2019; Lanferdini et al., 2020; Peyré-Tartaruga et al., 2021). It has been shown that the plantar flexors’ force generation has an important role in the body weight support, which, in turn, impacts C (Kram and Taylor, 1990; Teunissen et al., 2007). The triceps surae muscle-tendon unit (MTU) is very important for running performance, being responsible together with the quadriceps, for more than 70% of the total mechanical work during the stance phase (Sasaki and Neptune, 2006). It comprises three muscles (GM, gastrocnemius medialis; GL, gastrocnemius lateralis, SO, soleus) joined distally by a single tendon (i.e., the Achilles tendon). While GM and GL are biarticular muscles acting at both the knee and ankle joints, SO is monoarticular and acts only at the ankle joint. Their muscle architecture has implications for the force generated and transmitted to the tendon (Werkhausen et al., 2019) and, possibly, over C. In addition, MTUs with short fibers and long tendons (e.g., triceps surae) reduce C due to the lower muscle work required to perform terrestrial locomotion (Holt et al., 2014).

Furthermore, long-distance running consists of repetitive submaximal voluntary muscle contractions during successive steps. The force magnitude needed for optimal performance depends on the running velocity and on the lower limbs’ motion. Limb motion is determined by the muscle’s excursion (i.e., total length change during contraction) and shortening velocity. The higher the shortening velocity is, smaller is the generated force per muscle fiber, and recruitment of a larger number of muscle fibers is needed to improve performance, thereby increasing C. However, C of contraction can be minimized if muscle fascicles operate near the optimal length (Ishikawa et al., 2007; Lai et al., 2018) with a smaller excursion. There is evidence that, during the early stance phase of running, the triceps surae’s fascicles operate almost under isometric conditions (Ishikawa et al., 2007; Ishikawa and Komi, 2008; Lai et al., 2015, 2018). This suggests that a shorter number of sarcomeres per muscle fiber (i.e., shorter fascicles due to the shorter excursion needed), probably working at optimal length, are used during running. Therefore, muscles with shorter fascicle length (FL) and a low contraction velocity may result in a favorable contractile condition, once shorter FL can maintain tension with low activation energy cost (Fletcher and MacIntosh, 2017). Consequently, the length change of the triceps surae MTU mainly occurs in the tendon during the early stance phase of running (Lai et al., 2014, 2015), suggesting a high passive force production by the MTU elastic elements.

According to the literature, runners present a larger Achilles tendon cross-sectional area (CSA) and a higher Achilles tendon stiffness than non-runners (Rosager et al., 2002; Magnusson and Kjaer, 2003; Kongsgaard et al., 2005; Devaprakash et al., 2020), suggesting a tendon adaptation to accommodate this higher mechanical demand. Through this larger Achilles tendon CSA, the acting force is applied in a greater surface, thereby decreasing the tendon’s stress. Runners with smaller stress per limb cycle will have a greater possibility of storing elastic energy at their tendons during cyclic load, thereby reducing C and improving running performance. In addition, compared to non-runners, in the runners’ Achilles tendon, a greater amount of force will be required to stretch their tendon, which increases the amount of stored elastic strain energy (Rosager et al., 2002). The elastic storage capacity of a deformable structure is the area below the force-elongation curve, or the stress-strain curve, in which case the elastic energy is normalized by material volume. Elastic energy depends on MTU’s force and length limits and on the fixed or variable force-elongation curve slope, defined as stiffness (Pinto et al., 2021). Therefore, larger CSA tendons can store more elastic strain energy than thin tendons (minor CSA) at similar tendon elongation (Geremia et al., 2018) and, therefore, have higher stiffness. This is interesting as trained runners present higher Achilles tendon stiffness compared with healthy non-runners (Devaprakash et al., 2020). Furthermore, Pinto et al. (2021) found that the Achilles tendon material (maximum tangent modulus and stress) of elite long-distance runners (*N* = 6) is higher than that of pentathletes (*N* = 6). Moreover, the authors suggest that higher Achilles tendon stiffness is presumably associated with the highest weekly running training volume and with a tendency to reach higher elastic energy storage and release capacity. Finally, they found a significant negative correlation between running economy (RE) and Achilles tendon tangent modulus (*r* = −0.74), suggesting that long-distance runners can generate a greater running propulsion tendon force, enabling more RE and, possibly, greater storage and release of elastic energy (Pinto et al., 2021). These results agree with those found by Arampatzis et al. (2006) who found greater Achilles tendon force and stiffness in a group of endurance runners with better RE, compared to runners with lower RE, without differences on GM muscle architecture parameters. However, Devaprakash et al. (2020) demonstrated higher free Achilles tendon mechanical (stiffness), but not material properties (Young’s modulus) in runners (*N* = 16) compared with healthy non-runners (*N* = 16), whereas Rosager et al. (2002) did not find any difference in the Achilles tendon mechanical and material properties of runners [despite the small sample of runners (*N* = 5)] compared to non-runners (*N* = 10).

Assuming that shorter muscle FL improves C during running, as discussed above, a complementary adaptation that might occur to increase performance is an increase in tendon length. Ueno et al. (2018a) showed a significant positive correlation between Achilles tendon length [GM – myotendinous junction (MTJ)] and C during two submaximal running velocities (*r* ≥ 0.43), suggesting that longer Achilles tendon length may be advantageous to achieve superior running performance. Furthermore, Hunter et al.’s (2011) multiple linear regression suggested that longer lower limb tendons (especially the Achilles tendon) and less flexible lower limb joints are associated with improved RE. In contrast, Kovács et al. (2020) found no correlations between Achilles tendon length and marathon performance. Nevertheless, the authors concluded that the Achilles tendon CSA (*r* = 0.65) and SO physiological cross-sectional area (PCSA) (*r* = 0.77) and triceps surae PCSA (*r* = 0.72) are associated with better marathon performance (Kovács et al., 2020).

Additionally, SO encompasses a larger proportion of the triceps surae muscle volume and PCSA compared to the GM and GL, consequently being the strongest of the triceps surae muscles (Albracht et al., 2008). In addition, there is evidence that SO, probably, is the main plantar flexor muscle responsible for generating most of the mechanical work to lift and accelerate the body forward during the entire running stance phase (Hamner and Delp, 2013; Bohm et al., 2019, 2021b). However, SO has a higher percentage of slow-twitch fibers (86–89%; Johnson et al., 1973) compared to GM and GL. As the slow-twitch fibers are more economical/efficient at slow shortening velocities than the fast-twitch fibers (Barclay, 2015), SO probably contributes more to the C reduction compared to GM and GL. Furthermore, SO muscle fascicles shorten slower than the GM fascicles during early stance, reflecting their role as a crucial source of muscle force during running. Additionally, GM and GL exhibited delays in tendon recoil during the stance phase, reflecting their ability to transfer power and work between the ankle and the knee *via* tendon stretch and storage of elastic strain energy (Lai et al., 2018), differently from SO’s role as the main plantar flexor muscle in ankle propulsion. In addition, the mechanical interaction between SO and the Achilles tendon dynamically regulates the muscle fascicle shortening, allowing the decoupling of the fascicles (i.e., optimizes the operating fascicle velocity profile) in relation to the MTU due to the Achilles tendon lengthening and shortening, regulated by the tendon stiffness (Lai et al., 2016; Bohm et al., 2019, 2021a). This tendon decoupling mechanism allows a more economical condition to the muscle fascicles energy cost (i.e., reducing shortening cost and the active muscle volume) due to lower velocity and amount of shortening that is required from the muscle, resulting in a force generation improvement by the SO muscle fascicles, thus allowing energy savings (Cavagna et al., 1964; Kram, 2000; Fletcher and MacIntosh, 2015, 2017; Lai et al., 2016). Consequently, the mechanical work performed by the Achilles tendon reduces the quantity of mechanical work that would otherwise have to be performed by the triceps surae muscle (controlled by the Achilles tendon stiffness), explaining a reduction in C when the tendon is stretched and recoiled compared to if the muscle had to accommodate the lengthening/shortening alone (Bohm et al., 2021a).

The conflicting results found in the literature between triceps surae muscle architecture and/or Achilles tendon’s morphological, mechanical, and material properties’ independent variables, related to running performance, RE or, more specifically, to C, can be explained by the studies’ methodological differences regarding training characteristics (e.g., volume and intensity) and/or specificity of recruited sample size (Ooi et al., 2015; Pinto et al., 2021). Therefore, it is unclear which of the Achilles tendon parameters are associated with C. Similarly, although the triceps surae muscle generates tension actively, which suggests that the muscle is the dominant structure determining C (Kram, 2000; Fletcher and MacIntosh, 2017; Bohm et al., 2021a), we do not know which of the triceps surae muscle architecture parameters are related to C, and what is the relative contribution between the muscular and tendon parameters to C. Therefore, our goal was to determine whether triceps surae’s muscle architecture and Achilles tendon parameters are related to C in trained long-distance runners at different running speeds (12 and 16 km h^–1^). We hypothesize that SO muscle architecture parameters will be the main determinants of C. In addition, we hypothesized that the Achilles tendon’s morphological (i.e., tendon length or tendon CSA), mechanical (i.e., stiffness), and/or material (i.e., stress) properties will directly influence C.

## Materials and Methods

### Experimental Design

**FIGURE 1:**
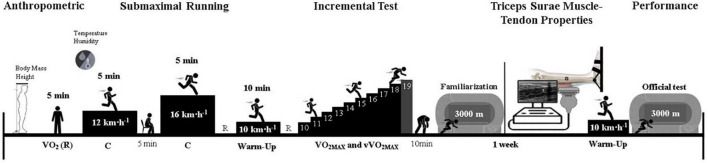
Experimental design. C, metabolic cost of running; 3,000-m, 3,000-m running performance; V̇O_2_, oxygen uptake; V̇O_2MAX_, maximal oxygen uptake; vV̇O_2MAX_, speed associated with maximal oxygen uptake; R, rest.

This study was characterized as a cross-sectional study. All participants were informed about the aims of the study, risks, and benefits of their participation in the study, and they gave their informed written consent to the experimental procedure, complying with the rules of the local scientific board. The study was approved by the Local Research Ethics Committee (No.: 2.437.616). The experimental procedures are shown in Figure 1. Each runner visited the laboratory on two occasions. In the first visit, anthropometry was evaluated. C was measured during submaximal steady-state running for 5 min at 12 and 16 km h^–1^. After 5 min, the maximal incremental test was performed. Then, after 10 min of rest, following the incremental test, the runners performed a familiarization with the athletics’ track to perform the 3,000-m running performance test. In the second visit, the triceps surae muscle-tendon’s morphological, mechanical, and material properties were assessed. After that, the 3,000-m running performance test was executed.

### Participants

Seventeen trained male recreational runners, all with at least 2 years of competitive running experience, all having regularly participated in regional and national middle and long-distance running competition events, with a training volume of at least 30-km⋅week^–1^ and reaching a minimum speed of 19-km h^–1^ during the incremental test were included in the present study (Table 1). Individuals with any medical restriction to the performance of maximal tests, any recent history of lower limb musculoskeletal injury, physical, cognitive, and/or psychological limitations to the execution and understanding of the tests were excluded. All the runners were instructed to not engage in any vigorous physical activity for 48 h before the tests (Baroni et al., 2013).

**TABLE 1:** Sample characterization in mean, standard deviation (SD), and confidence interval (CI).

Parameters	*M* *e* *a* *n*	SD (±)	CI (95%)
Age (years)	34.2	7.4	30.4–38.1
Body mass (kg)	67.5	6.7	64.0–70.9
Height (cm)	173.1	6.8	169.6–176.6
C12 (J kg^–1^m^–1^)	3.66	0.26	3.52–3.79
C16 (J kg^–1^m^–1^)	3.66	0.20	3.56–3.76
V̇O_2MAX_ (ml kg^–1^min^–1^)	62.5	6.2	59.3–65.7
vV̇O_2MAX_ (km h^–1^)	19.8	1.5	19.0–20.5
3000 m (s)	649.9	48.8	624.8–675.0

*V̇O_2MAX_, maximal oxygen uptake; vV̇O_2MAX_, speed associated with maximal oxygen uptake; C12, metabolic cost at 12 km h^–1^; C16, metabolic cost at 16 km h^–1^; 3,000-m, 3,000-m running performance.*

### Data Collection

#### Anthropometric Evaluation

Anthropometry was evaluated according to the International Society for Advancement of Kinanthropometry (Marfell-Jones et al., 2012). The athletes’ body mass (kg) was measured with a portable electronic scale (Model UP-150, Urano, Brazil) with a 100 gresolution. Their height was measured with a tape measure (Simple Fiber Tread, Sanny, Brazil) with 1 mm of resolution.

#### Submaximal Running Tests

In the first visit, the oxygen uptake (V̇O_2_) was collected at rest in the standing position for 5 min. Then, the athletes performed a familiarization trial on a motorized treadmill (Super ATL, Inbrasport/Inbramed, Porto Alegre, Brazil), consisting of a 10-min warm-up run at 10 km h^–1^. Next, the submaximal tests were performed for 5 min at 12 and 16 km h^–1^ (approximately 58.5 and 78.1% of the maximal incremental test speed, respectively), with a 5-min rest between tests (Saunders et al., 2004). The treadmill speeds were calibrated before each test (Mocap System). The breath-by-breath V̇O_2_ was measured using an open-circuit indirect calorimetry system (Cosmed, Quark CPET, Rome, Italy).

#### Maximal Incremental Running Test

The maximal incremental running test started at a 10-km h^–1^ speed, which was increased by 1 km h^–1^ every minute until volitional exhaustion (Bentley et al., 2007). The V̇O_2_ was measured as described in the submaximal running test. All runners reached the volitional exhaustion, plateau in the V̇O_2_ course and/or the respiratory exchange ratio above 1.15 in the end of the test.

#### Triceps Surae Muscle Architecture

A B-mode ultrasonography system (LOGIQ P6, GE Healthcare, Waukesha, WA, United States) and a linear array matrix transducer (50 mm, 15 MHz - GE Healthcare, Waukesha, WA, United States) were used to determine FL, pennation angle (PA), and muscle thickness (MT) of GM, GL, and SO, respectively, in the dominant leg. The frequency was set to 9 MHz, the depth to 6 cm. The triceps surae muscle architecture images were obtained with the subjects in the ventral decubitus, with the knees fully extended (0°) and the ankle in neutral position (foot plantar surface perpendicular to the shank) at rest. The ankle joint position was controlled using a digital goniometer (DIGIMESS, United States).

Three ultrasound images were obtained for each muscle. The ultrasound probe was positioned longitudinally to the muscle fibers and perpendicular to the skin at 30% (GM, GL) and 50% (SO) of the distance between the popliteal fossa and the lateral malleolus (Kawakami et al., 1998; Geremia et al., 2019). The ultrasound probe was covered with water-soluble transmission gel. Probe alignment was considered appropriate when superficial and deep aponeuroses were parallel, and several fascicles could be easily delineated without interruption across the image (Baroni et al., 2013).

#### Achilles Tendon’s Morphological Properties

Achilles tendon’s morphological properties were determined from ultrasound images (Muramatsu et al., 2001; Arya and Kulig, 2010; Geremia et al., 2018). The images were collected by the same ultrasound system and with the participant positioned as described previously in the triceps surae muscle architecture evaluation.

Tendon length was obtained from longitudinal images of the Achilles tendon. The most distal point of the tendon on the calcaneus in a sagittal view was identified on the ultrasound images, and the respective point was marked on the skin. GM’s MTJ was identified by moving the transducer proximally, and this point was marked on the skin. The distance between these two points marked on the skin was measured using a tape measure and represented tendon length at rest (Arya and Kulig, 2010; Geremia et al., 2018).

Achilles tendon CSA was obtained with the ultrasound probe positioned perpendicular to the tendon, with the subject in the prone position. Three CSA images were obtained at 2, 4, and 6 cm from the Achilles tendon insertion at the calcaneus (Arya and Kulig, 2010; Geremia et al., 2018).

#### Achilles Tendon’s Mechanical and Material Properties

Tendon force-elongation and stress-strain relationships were determined by maximal voluntary isometric contractions (MVIC) on an isokinetic dynamometer (Biodex System 3 Pro, 2000 Hz, Biodex Medical Systems, United States). A video camera (HDR-CX, 60 Hz, Sony, Japan) was synchronized with the isokinetic dynamometer using a LED light signal captured by a video camera and a square-wave electric signal triggered into a data acquisition system (Windaq data collection system, DATAQ Instruments, Akron, 16-bit, United States), and ultrasound images were assessed by an external DVD recording unit (R130/XAZ, 32 Hz, Samsung Seoul Inc., South Korea), with a sampling frequency of 30 frames per second. A synchronization system (HORITA, Video Stop Watch VS-50; HORITA Company Inc., United States) was triggered to add a time frame to the ultrasound video recording system, thereby allowing the synchronization of the ultrasound video and images, torque, muscle activation, and kinematic parameters.

#### Tendon Elongation During Plantar Flexor Ramp Contractions

Tendon elongation was determined during plantar flexor contractions, with the participants seated on the isokinetic dynamometer with the hip flexed at 85° (0° = the hip fully extended), the knee fully extended, and the ankle in neutral position. The plantar/dorsiflexion axis was aligned with the dynamometer’s axis of rotation, and the foot was fixed on it by velcro straps, which were also used to fix the leg on the dynamometer chair and stabilize the trunk and hips. The ultrasound probe was positioned at the GM’s MTJ, longitudinally to the tendon’s mechanical axis. A skin marker (masking tape) positioned under the ultrasound probe during the MVICs was used to detect possible movements of the ultrasound probe over the skin. When any movement was detected, tendon elongation corrections were carried out according to the magnitude of the ultrasound probe movement relative to the skin.

The participants performed three ramped plantar flexor MVICs as familiarization and tendon preconditioning (Geremia et al., 2018). Then, subjects performed two ramped plantar flexor MVICs lasting 10 s each (Magnusson et al., 2001; Geremia et al., 2018), with 2-min intervals between contractions. During the plantar flexor MVICs, torque, ultrasound video, tibialis anterior (TA) muscle activation, and ankle kinematics (see below) were recorded. The MVIC with the highest peak torque was used for data analysis.

#### Plantar Flexor Torque Correction Through Muscle Electrical Activity Evaluation

The torque recorded by the dynamometer corresponded to the final torque of the 10-s plantar flexion MVIC. Corrected plantar flexion torque was determined for foot gravitational torque using predicted weight, as described in the inertial parameters table of de Leva (1996) and for dynamometer gravitational torque. However, in the presence of antagonistic muscle co-activation, the torque generated during plantar flexion is smaller than the maximal torque produced by the plantar flexors (Magnusson et al., 2001; Geremia et al., 2018). For this reason, the plantar flexion torque was corrected through the relationship between the TA electromyography signal and the corresponding dorsiflexion torque (Arya and Kulig, 2010; Waugh et al., 2012).

Tibialis anterior activation was obtained by surface electrodes (bipolar configuration; 20-mm interelectrode distance; Ag/AgCl, Meditrace, Kendall, Canada) in three different conditions (Mademli et al., 2004; Geremia et al., 2018): (a) at rest, (b) at a lower, and (c) at a higher activation than that produced during the ramp plantar flexion contractions. The electromyography signals were amplified (AMT-8, Bortec Biomedical Ltd., Canada) and registered simultaneously with the dorsiflexion torque performed on the dynamometer. The electromyography signals were band-pass filtered (20–500 Hz), rectified, and smoothed with a low-pass filter (Butterworth, 4 Hz). A linear relationship was established between the electromyography signal and the obtained dorsiflexion torque at the three different activation levels. This allowed the estimation of the antagonistic torque during the MVIC (Mademli and Arampatzis, 2005; Geremia et al., 2018). From this relation, the torque estimated during co-activation was added to the torque measured by the dynamometer during MVIC, thereby correcting the maximal plantar flexion torque.

#### Tendon Displacement Correction Through Evaluation of Ankle Joint Rotation

Regardless of segments external fixation during MVICs, joint rotations can occur, causing undesired ankle plantar flexion during maximal effort, overestimating tendon elongation (Magnusson et al., 2001). Therefore, reflective markers were placed at the middle third of the leg, the malleolus, the hallux, the calcaneus, and the upper and lower extremities of the isokinetic dynamometer footplate to correct for this effect. These markers’ position was monitored by a video camera during MVICs (Muramatsu et al., 2001).

A two-segment bidimensional planar model (foot = hallux marker, calcaneus marker, and malleolus marker; leg = leg marker to malleolus marker) was used to calculate the plantar flexion angle at rest and during the ramp contraction up to MVIC. Achilles tendon displacement was corrected after tracking the GM’s MTJ during ankle passive motion (Magnusson et al., 2003; Geremia et al., 2018). The passive movements were performed at a constant angular speed of 5 degrees⋅s^–1^ from 90 degrees (tibia perpendicular to the foot line with the knee fully extended) to 125 degrees. Three passive plantar flexion cycles were performed for the analysis. If the electromyography activity from GM, SO, and TA was detected visually on the computer screen, the cycles were invalidated and the trial was repeated.

Gastrocnemius medialis MTJ tracking was performed using the SkillSpector software (1.3.2, Video4Coach, Denmark). MTJ displacement was obtained for each joint angle in the three passive movement cycles. Therefore, the mean displacement from each joint angle was used for the analysis. The MTJ displacement obtained during MVICs was corrected by subtracting the MTJ displacement caused by joint rotation (Muramatsu et al., 2001; Magnusson et al., 2003; Geremia et al., 2018).

#### 3,000-m Running Performance

After evaluations of muscle architecture and tendon properties in the laboratory, the athletes went to the outdoor 400-m official athletics track and performed a warm-up for 10 min. After warming up, the runners performed the 3,000-m running performance test. Two digital stopwatches (Timex T5K 491Sr/Ti, United States) were used to measure the test time for each athlete by two researchers positioned at the start/finish line. The 3,000-m running performance test time was obtained by the average time between the two evaluators. At every 1,000 m and in the last 400 m, all the athletes were verbally encouraged to run at their best time.

### Data Analysis

#### Metabolic Cost

The breath-by-breath V̇O_2_ was obtained for all the athletes and averaged during the last 2 min of each running speed (12 and 16 km h^–1^). C was calculated in each trial speed using the equation proposed by Péronnet and Massicotte (1991) and expressed in J kg^–1^m^–1^ (di Prampero et al., 1986; Peyré-Tartaruga et al., 2021). The respiratory exchange ratios (V̇CO_2_/V̇O_2_) remained at <0.95 for all trials.

#### Maximal Incremental Running Test

The breath-by-breath V̇O_2_ values were averaged in the last minute of each speed to exclude values with four standard deviations above or below the average of the moving windows of the entire curve. An average of three breaths was obtained in each window (Fernandes et al., 2012). V̇O_2MAX_ was determined as the highest value analyzed in the last test stage (Bentley et al., 2007).

#### Triceps Surae Muscle Architecture

All ultrasound images were analyzed by the same investigator with extensive experience using ultrasonography analysis software (Image J software, version 1.48v, National Institutes of Health, Bethesda, MD, United States). The best fascicle (i.e., the fascicle that could be seen in its entirety from its insertion on the deep aponeurosis into the superficial aponeurosis, or to the ultrasound probe field-of-view end) in each image was used for muscle architecture analysis (Geremia et al., 2019). FL was considered the length of the fascicular path in a straight line between superficial and deep aponeuroses. When the ends of the fascicles were outside the ultrasound image, FL was estimated from extrapolation as recommended in previous study (Finni and Komi, 2002). PA was calculated as the angle between the muscle fascicle and the deep aponeurosis. Mean values were obtained from three ultrasound images for each triceps surae muscle to determine FL and PA (Geremia et al., 2019). Despite that, error in estimating the entire FL using the linear model was 2–7% described by Finni and Komi (2002) and 13% by Noorkoiv et al. (2010).

#### Achilles Tendon’s Morphological Properties

All ultrasounds’ images were analyzed with an open-source image processing program (ImageJ software, version 1.48v; National Institutes of Health, Bethesda, MD, United States). The tendon CSA was manually outlined (excluding the paratenon) and calculated by the software. Achilles tendon CSA was measured five times during rest in each ultrasound image, and the mean value was determined for each position. The mean value from the three positions was taken as the Achilles tendon CSA (Arya and Kulig, 2010; Geremia et al., 2018). Achilles tendon length was measured as previously described in the data collection, following previous procedures in the literature (Arya and Kulig, 2010; Geremia et al., 2018).

#### Achilles Tendon’s Mechanical and Material Properties

Raw data from passive and active dynamometry assessments were processed in LabVIEW (version 15.0, National Instruments). Achilles tendon force was estimated by the ratio between the corrected plantar flexion torque and the Achilles tendon moment arm. Following previous studies, the moment arm was taken to be 11% of lower leg length, determined as the distance from the malleolus lateralis to the articular cleft between the femur and tibia condyles (Greive et al., 1978; Kubo et al., 2005; Muraoka et al., 2005). Tendon elongation was obtained from the MTJ displacement corrected during the ramp MVIC. Virtual Dub software (Avery Lee, United States) was used to screen the desired images frame by frame. After selecting the images, the MTJ displacement was determined by the SkillSpector software (1.3.2, Video4Coach, Denmark). Achilles tendon force and elongation were estimated at 10% intervals up to MVIC (from 0 to 100%). The slope of the force-elongation curve in linear function obtained between 50 and 100% of the MVIC was considered as the Achilles tendon stiffness (Geremia et al., 2018).

Stress was obtained by the ratio between tendon force and the Achilles tendon CSA, while strain by the ratio between tendon elongation at MVIC and tendon length was at rest. The stress and strain values were obtained at intervals of 10% of MVIC (from 0 to 100%) (Geremia et al., 2018). Young’s modulus was determined as the slope of the stress-strain curve in linear function between 50 and 100% of the maximal stress (Geremia et al., 2018). The slope of these curves between 50 and 100% was obtained by linear regressions.

### Statistical Analysis

The data are presented as mean and standard deviation, and 95% confidence intervals. Data normality was evaluated using the Shapiro—Wilk test. Stepwise multiple linear regression was used to determine the relationship between independent variables [Stiffness (N/mm), Young’s modulus (MPa), stress (MPa), strain (%), CSA (mm^2^), tendon length (mm), force (N), FL of GM, GL, and SO (mm), and PA of GM, GL, and SO (°)], and the dependent variables C12 (J kg^–1^m^–1^) and C16 (J kg^–1^m^–1^). Our collinearity diagnostic exploration resulted in variance inflation factors (VIF) of <2, which indicates acceptable multicollinearity levels of the variables (Dormann et al., 2013; Batterson et al., 2020). In addition, *post hoc* power of the multiple linear regressions was calculated according to Cohen et al. (2002). *A priori* effect size (ES) of multiple linear regression was calculated and classified as small (>0.02); moderate (>0.13); and large (>0.26), using G*Power 3.1.9.7 (FrauzFaurUniversitat Kiel, Germany), described by Faul et al. (2009). The Pearson’s correlation coefficient was classified as small (*r* ≥ 0.1); moderate (*r* ≥ 0.3); large (*r* ≥ 0.5); very large (*r* ≥ 0.7); and extremely large (*r* ≥ 0.9) according to thresholds recommended by Hopkins et al. (2009), using RStudio (R version 4.1.0, R Core Team, 2021). All statistical tests were performed using the statistical software (SPSS 20.0 for Windows, IBM, Chicago, United States), with an *a priori* significance level of α ≤ 0.05.

## Results

Table 2 presents muscle architecture parameters and Achilles tendon’s morphological, material, and mechanical properties of the participants.

**TABLE 2:** Muscle architecture parameters and Achilles tendon’s morphological, mechanical, and material properties in mean, standard deviation (SD), and confidence interval (CI).

	Mean	SD (±)	CI (95%)
**Muscle fascicle length**			
GM (cm)	4.54	0.92	4.06–5.01
GL (cm)	5.33	1.55	4.53–6.12
SO (cm)	4.18	0.56	3.89–4.46
**Muscle pennation angle**			
GM (°)	24.36	3.32	22.66–26.07
GL (°)	15.29	4.51	12.98–17.61
SO (°)	23.73	1.40	23.01–24.45
**Tendon morphological properties**			
CSA (mm^2^)	52.6	10.1	47.4–57.8
TL (mm)	195.3	17.5	186.2–204.3
**Tendon mechanical properties**			
Force (N)	3255.2	671.9	2909.8–3600.7
Elongation (mm)	22.1	5.7	19.1–25.0
Stiffness (N/mm)	156.7	50.3	130.9–182.4
**Tendon material properties**			
Stress (MPa)	64.1	18.1	54.8–73.4
Strain (%)	11.4	2.8	9.90–12.8
Young’s modulus (MPa)	567.6	160.1	485.3–649.9

*GM, gastrocnemius medialis; GL, gastrocnemius lateralis; SO, soleus; CSA, Achilles tendon cross-sectional area; TL, tendon length.*

As shown in Table 3, 69% of C can be explained together by negative relation with SO PA and Achilles tendon CSA at 12 km h^–1^ [*F*(2,14) = 15.707; *p* < 0.001; *r*^2^ = 0.691; *ES* = 2.24; and observed power = 0.28], whereas 63% of C can be explained together by negative relation with SO PA and positive relation with Achilles tendon stress at 16 km h^–1^[*F*(2,14) = 12.001; *p* = 0.001; *r*^2^ = 0.632; *ES* = 1.75; and observed power = 0.23].

**TABLE 3:** Determinants of metabolic cost at 12 km h^–1^ (C12) and at 16 km h^–1^ (C16) of running on a treadmill.

Dependent variable	*R* ^2^	*P*	Indicator	Standardized coefficients (β)	*P*
C12 (J kg^–1^m^–1^)	0.691	<0.001	CSA (mm^2^)	–0.517	0.004
			SO PA (°)	–0.628	0.001
C16 (J kg^–1^m^–1^)	0.632	=0.001	SOPA (°)	–0.582	0.003
			Stress (MPa)	0.586	0.003

*CSA, Achilles tendon cross-sectional area; SO PA, soleus pennation angle.*

The equation used to predict C (negatively to SO PA and CSA) at 12 km h^–1^ (C12) was:

C⁢12=7.141+(-0.013)⁢C⁢S⁢A+(-0.117)⁢S⁢O⁢P⁢A

where C12 is the running metabolic cost at 12 km h^–1^ (J kg^–1^m^–1^), CSA is the Achilles tendon cross-sectional area (mm^2^), and SO PA is the soleus pennation angle (°).

The equation used to predict C (negatively to SO PA and positively to stress) at 16 km h^–1^ (C16) was:

C⁢16=5.187+(-0.082)⁢S⁢O⁢P⁢A+(0.006)⁢stress

where C16 is the running metabolic cost at 16 km h^–1^ (J kg^–1^m^–1^) and SO PA is the soleus pennation angle (°) and stress is measured in MPa.

**FIGURE 2:**
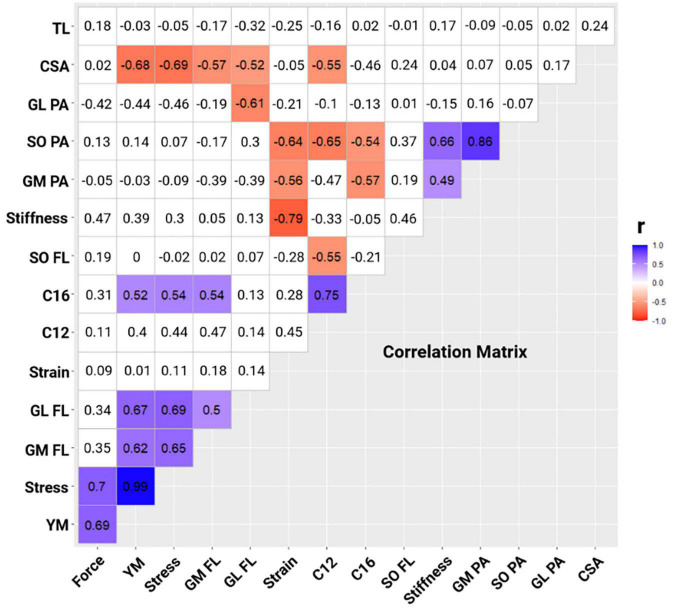
Correlation matrix between metabolic cost- C [C12 at 12 km h^–1^; C16 at 16 km h^–1^] and plantar flexor muscle-tendon properties [CSA, Achilles tendon cross-section area; GL FL, gastrocnemius lateralis fascicle length; GL PA, gastrocnemius lateralis pennation angle; GM FL, gastrocnemius medialis fascicle length; GM PA, gastrocnemius medialis pennation angle; SO FL, soleus fascicle length; SO PA, soleus pennation angle; TL, tendon length; YM, Young’s modulus]. Blue cells show a significant positive correlation between the variables; red cells show a significant negative correlation between the variables (*p* < 0.05); and white cells show non-significant correlations between the variables (*p* > 0.05).

Stepwise multiple linear regression analyses limited initial values included in the model as per correlational collinearity (VIF > 2) presented between SO PA and GM PA (*r*^2^ = 0.743) or stiffness (*r*^2^ = 0.441), and between tendon stress and Young’s modulus (*r*^2^ = 0.989). Correlation matrix (Figure 2) showed the magnitude of Pearson’s product-moment correlation coefficient between C and triceps surae muscle-tendon properties [blue (positive) and red (negative)]. Therefore, it can be observed (Figure 2) that the independent variables SO PA (*r* = −0.65), SO FL (*r* = −0.55), and Achilles tendon CSA (*r* = −0.55) have a large negative correlation with the dependent variable C12. Similarly, the independent variables GM PA (*r* = −0.57) and SO PA (*r* = −0.54) presented a negative correlation, while GM FL (*r* = 0.54), stress (*r* = 0.54), and Young’s modulus (*r* = 0.52) presented large positive correlations with the dependent variable C16 (Figure 2).

## Discussion

The purpose of this study was to determine whether triceps surae’s muscle architecture (FL and PA of GM, GL, and SO) and Achilles tendon parameters (morphological, mechanical, and material properties) are related to C in trained long-distance runners at different running speeds (12 and 16 km h^–1^). According to our results, 69% of C was probably determined by SO PA and Achilles tendon CSA at 12 km h^–1^, whereas 63% of C was probably determined by SO PA and Achilles tendon stress at 16 km h^–1^. Therefore, these results demonstrate that, among the muscle architecture and Achilles tendon parameters, only SO PA, Achilles tendon CSA, and stress play an important role in C at different running speeds (12 and 16 km h^–1^). Despite the large ES of our results from the stepwise multiple linear regression model, results showed small observed power due to the low sample size, limiting the cause-effect of the regression analysis. Additionally, our results showed a large negative correlation between SO PA, SO FL, and Achilles tendon CSA with C12 (Figure 2). Furthermore, C16 presented a large negative correlation with GM PA, SO PA, and GM FL, and a large positive correlation with stress and Young’s modulus (Figure 2).

Our results demonstrated that runners with greater SO PA, GM PA (collinearity with the SO PA), larger Achilles tendon CSA and/or lower stress, and Young’s modulus (collinearity with the stress) presented lower C. Muscles with short fibers and high PA (e.g., plantar flexors) and long compliant in-series tendons are well suited for economical energy cost during locomotion, once elastic tendons store and return mechanical energy over each step (Biewener and Roberts, 2000; Roberts, 2002; Roberts and Azizi, 2011). Apparently, short-fascicled muscles (i.e., with smaller serial sarcomere numbers) can reduce the energy consumption needed (Adenosine Triphosphate - ATP) per unit force generated compared to muscles with longer fascicles (Roberts et al., 1998; Fletcher and MacIntosh, 2017). Consequently, a muscle with longer fascicles will involve a greater active muscle volume, and, therefore, a greater amount of ATP will be consumed (Fletcher and MacIntosh, 2017). An increase in SO PA is usually related to a parallel sarcomere (and, therefore, force production capacity) increase in muscle fibers due to training. However, this increased PA may reduce the total fascicle shortening (i.e., a smaller fascicle excursion), limiting the muscle length for force production and reducing its shortening velocity. A higher muscle force production capacity means that a higher mechanical load will be transferred to the tendon, thereby affecting the tendon structure (e.g., increasing tendon CSA). The fact that Achilles tendon CSA has been found as a determinant of C is probably related to its higher capacity of storing and releasing elastic energy, which enables runners to run more efficiently (Kovács et al., 2020).

According to the literature, the plantar flexor muscles strength and Achilles tendon stiffness (collinearity with the SO PA in our results) directly influence C (Arampatzis et al., 2006; Albracht and Arampatzis, 2013). A stronger muscle with shorter fascicles will demand a higher tendon mechanical capacity (i.e., higher stiffness), which is probably related to an improvement in the operating fascicle force-velocity profile, mainly during the tendon lengthening, improving the muscular work production efficiency (Bohm et al., 2021a). A stiffer Achilles tendon may reach higher elastic energy storage and release capacity (Pinto et al., 2021). As the runners present similar Achilles tendon strain during running compared to the non-runners and the runners with different running economies (Rosager et al., 2002; Arampatzis et al., 2006), a larger Achilles tendon CSA will require a greater amount of force to stretch the tendon, increasing the elastic strain energy stored (Rosager et al., 2002). Assuming that SO generates greater force compared to GM and GL, then SO can be the main contributor to the elastic strain energy storage in the Achilles tendon (Lai et al., 2014, 2018). Our results may help to explain which structural parameters are responsible for decreasing the muscle energy cost. Furthermore, the ability of pennate muscles (e.g., triceps surae) to undergo dynamic changes (e.g., running) presents significant interactions with muscle architectural gearing during contractions (Eng et al., 2018). Muscle architectural gearing is usually considered a mechanism to reduce a muscle metabolic energy consumption, allowing slower muscle fascicle contractions (i.e., the belly shortening more than the fascicle) (Azizi et al., 2008; Wakeling et al., 2011; Bohm et al., 2021b). At the same time (and often neglected), a greater PA also means that the muscle fascicle forces are less aligned with the effective muscle force (i.e., the muscle belly’s line of action). However, muscles with greater PA may operate with higher belly gearing, which is associated with higher strength potentials (Monte et al., 2021), and consequently present higher relation with C (Bohm et al., 2019). The increase in the muscle force potential due to the reduction in the contraction velocity (i.e., more pennate fascicles have less serial sarcomeres and thereby smaller shortening velocity) possibly outweighs the decrease in muscle’s effective force production (Azizi et al., 2008; Wakeling et al., 2011). Finally, pieces of evidence that C is related to SO (and, to a lesser extent, to GM) (Lai et al., 2016; Bohm et al., 2019; Werkhausen et al., 2019) demonstrate that these lower muscle fascicle contraction velocities (i.e., higher force-velocity potential) contribute significantly during the running propulsive phase. However, our study did not investigate this dynamic relationship, only evaluating the plantar flexors’ muscle architecture at rest.

Additionally, the results of the present study showed that larger Achilles tendon CSA and/or lower tendon stress and Young’s modulus (collinearity with the stress) determine lower C. Achilles tendon’s morphological characteristics can also decrease the contractile elements’ C (Magnusson and Kjaer, 2003; Ueno et al., 2018a,b). Our results (Figure 2) partially agree with Kovács et al. (2020), who found a large correlation between the Achilles tendon CSA and running performance. However, findings from Pinto et al. (2021) are not in line with our results. While our results showed a large positive correlation between Young’s modulus and C at 16 km h^–1^, the results of Pinto et al. (2021) demonstrate a very large negative correlation between tangent modulus and RE at 16 km h^–1^. According to Pinto et al. (2021), elite runners may generate a greater tendon force at the propulsion phase of running, thereby resulting in a more economical energy use and reducing RE during running. However, discrepancies with our results may be associated with the different levels of athletes between studies. While we evaluated endurance recreational runners, Pinto et al. (2021) evaluated endurance elite runners. In addition, differences between Achilles tendon’s material properties measuring methods, training characteristics (e.g., volume) and specificity of sample size recruited contributed to these between-studies differences (Ooi et al., 2015; Pinto et al., 2021). Our results are in agreement with those of Albracht and Arampatzis (2013), who found an increase in maximum plantar flexion muscle strength and Achilles tendon stiffness after 14 weeks of strength training, which enhanced RE (∼4%) without changes in the other investigated variables (e.g., GM PA, GM FL, and Achilles tendon length). In addition, our results demonstrate a large correlation between SO PA (12 and 16 km h^–1^) and GM PA (16 km h^–1^) with the C, corroborating the results of Kovács et al. (2020), who found a very large correlation between SO PCSA and triceps surae PCSA with better marathon performance. Furthermore, it is noteworthy that our results were built from a stepwise multiple linear regression model, where the determinants SO PA and Achilles tendon CSA (69%) or stress (63%) predicted the C at submaximal running speeds at 12 and 16 km h^–1^, respectively. Furthermore, the runners present significantly larger Achilles tendon CSA than the non-runners (Rosager et al., 2002; Magnusson and Kjaer, 2003; Kongsgaard et al., 2005), which, in turn, could lead to a lower C due to the lower stress, thereby suggesting a better running performance (Rapoport, 2010). Rosager et al. (2002) have shown a reduction in tendon stress for a similar tension in the runners with larger Achilles tendon CSA compared to the non-runners. They suggested that, although a similar Achilles tendon strain occurs during running, a larger Achilles tendon CSA is associated with higher tendon stiffness and a better ability to store and release elastic strain energy (Rosager et al., 2002; Monte et al., 2020). Arampatzis et al. (2006) found a higher triceps surae contractile strength and higher tendon stiffness in the most economical runners, without differences in the lower limbs’ kinematic characteristics of least economical runners, which suggests a similar tendon strain between groups once the fascicles do not change their excursion with increasing tendon stiffness. Therefore, a larger Achilles tendon CSA allows a better force distribution by the tendon area during each running step, protecting the tendon against overloading through a tendon stress reduction for the same elongation (Rosager et al., 2002; Kongsgaard et al., 2005, 2007), improving the muscle-tendon force transmission and running economy, thereby reducing C.

However, other pieces of evidence showed a negative correlation between Achilles tendon length and C (Hunter et al., 2011; Ueno et al., 2018a; Monte et al., 2020). According to these studies, the longer Achilles tendon length can store and release more elastic strain energy than shorter tendons, being considered the most important tendon’s morphological factor in achieving better running performance (Hunter et al., 2011; Ueno et al., 2018a). However, our results do not support this idea as we did not find correlations between tendon length and C (see Supplementary Material). In addition, these previous studies (Hunter et al., 2011; Ueno et al., 2018a; Monte et al., 2020) found no correlation between Achilles tendon CSA and C, which is also contrary to our observations. Monte et al. (2020) demonstrated that the faster runners (maximal treadmill running speed) presented lower C, and the faster half-marathon pace of these runners was associated with the larger Achilles tendon CSA. Our study results showed that the Achilles tendon CSA, together with SO PA, determined 69% of C.

## Limitations

The main limitation of the present study was that it neglected the Achilles tendon curvature. However, neglecting the Achilles tendon curvature led to a small overestimation (<2 mm) of the Achilles tendon moment arm (Obst et al., 2017). Furthermore, in the present study, only maximal isometric ramp contractions of the plantar flexors were performed, which may have minimally modified the Achilles tendon curvature, and limited ankle rotations (∼13 degrees) are present during these contractions (Arampatzis et al., 2008). In addition, recently, Buffey et al. (2021) have evaluated the Achilles tendon moment arm with three different measurement methods [magnetic resonance imaging (MRI); tendon excursion (TE); and dual-energy X-ray absorptiometry (DXA)]. Buffey et al. (2021) determined the Achilles tendon moment arm values at 0° ankle joint angle (MRI: 43.3 mm; TE: 29.5 mm; and DXA: 53.8 mm) and found a variation between 43.3 and 45.4 mm (ankle joint angles of 0° and 10° of plantar flexion, respectively) for the Achilles tendon moment arm measured by MRI. Our results (45.7 ± 2.2-mm Achilles tendon moment arm), measured indirectly (0° ankle joint), showed similarities with Buffey et al.’s (2021) moment arm measured by MRI in subjects with similar height. Furthermore, DXA’s Achilles tendon moment arm measures were significantly greater than the MRI measurements (19.7–24.9%) and were 45.2% significantly larger than the TE method (Buffey et al., 2021), demonstrating that, regardless of the method used, all methods have limitations for measuring the moment arm compared with the MRI method. Although different methods of Achilles tendon moment arm measurement have limitations, this limitation should not prevent them from being used in different studies (Seynnes et al., 2015; Buffey et al., 2021). Additionally, the entire data collection and analysis procedures were already well established by previous studies (Arampatzis et al., 2006; Geremia et al., 2018; Bohm et al., 2021a,b), and the possible Achilles tendon moment arm error was systematic for all investigated subjects. Moreover, as tendon force is calculated from the moment arm, a mismatch between the assumed moment arm and the actual moment arm can affect the calculated force and, subsequently, the tendon stress and stiffness (Hashizume et al., 2016). The present study did not consider the elastic strain energy storage and release in the Achilles tendon. These parameters are associated with the energy conversion in the series elastic element, which can differentiate running economies from runners with similar physiological parameters (Arampatzis et al., 2006) and could be a potential C determinant. In addition, we did not measure the MTU behavior dynamically, using technological devices during running (e.g., ultrasonography of the muscle tendon), which would allow us to better understand the determinants of the C behavior during each running step (Bohm et al., 2021a,b). Another limitation of this study was to measure the Achilles tendon CSA by ultrasound at three different distances (2, 4, and 6 cm) from the Achilles tendon insertion at the calcaneus (Arya and Kulig, 2010; Geremia et al., 2018). Achilles tendon presents different CSA values in its proximal and distal portions, and there are specific portions to better estimate the Achilles tendon CSA through MRI, considered a precise (Magnusson and Kjaer, 2003; Kongsgaard et al., 2005) and, perhaps, better tool than ultrasound. Bohm et al. (2016) suggested that the ultrasound-based methodology is not recommended for an accurate Achilles tendon CSA determination *in vivo* compared to the MRI. However, we did not have access to MRI equipment, and, therefore, we measured the muscle-tendon properties through ultrasound-based methodology, which, despite the limitations, is similar to several other studies (Arampatzis et al., 2006; Arya and Kulig, 2010; Geremia et al., 2018; Bohm et al., 2021a,b). Another limitation was the non-evaluation of the triceps surae muscles’ PCSA (Kovács et al., 2020). We did not have equipment available for measuring PCSA. Greater PA is usually found in muscles with greater PCSA by accommodating a greater CSA of muscle fascicles attaching to a tendon (i.e., more fascicles packed into an area). In addition, the ultrasound measurement of the GM, GL, and SO was standardized at 30% (GM, GL) and 50% (SO) of the distance between the popliteal fossa and the lateral malleolus according to the literature (Kawakami et al., 1998; Geremia et al., 2019) and not standardized to the muscle length. Therefore, these measurements can show us different relative positions along the length of each muscle, and the muscle architecture (i.e., FL and PA) presented in this study could be a reflection of the different muscle sites. Finally, this study looked at associations between many variables together; therefore, causal links between Achilles tendon CSA, stress, and SO PA with C12 and C16 cannot be established appropriately. Additionally, our results showed large ES and small observed power of the stepwise multiple linear regression model, probably due to the low sample size, which consequently limits precision of the cause-effect on stepwise multiple linear regression results. Therefore, we suggest that future studies have sample sizes corresponding to two times the number of independent variables used in multiple regression models.

## Future Perspectives

Future longitudinal studies should examine whether training-induced changes in the Achilles tendon’s mechanical, morphological, and material properties, as well as in the muscle architecture parameters, are associated with the predicted changes in C found in our study. Also, future studies should analyze different running performance distances (e.g., 5,000 m, 10,000 m, half marathon, and marathon) to better understand the relationships between C and Achilles tendon’s morphological properties (e.g., CSA, length) found in the literature (Ueno et al., 2018a; Kovács et al., 2020; Monte et al., 2020), as well as with the muscle architecture (e.g., PA, FL). In addition, analyzing the muscle-tendon properties during running may give us new perspectives on C and running performance. Additionally, we also suggest that other statistical models (i.e., non-linear cubic regression; quadratic regression; or Bayesian regression) be used to investigate the relationship between muscle tendon’s morphological and mechanical properties with C and running performance.

## Practical Applications

Understanding the muscle-tendon variables, determinants of C and running performance in a laboratory setting may contribute to improve prescription of specific exercises by sports scientists, coaches, and athletes looking to improve the Achilles tendon properties and triceps surae muscle architecture, thereby improving running performance in long-distance runners.

## Conclusion

In summary, stepwise multiple linear regression results demonstrated that C in trained recreational long-distance runners is related (63–69%) to muscle-tendon characteristics of the plantar flexors [e.g., negative relation with SO PA, GM PA (collinearity with the SO PA) and Achilles tendon CSA, and positive relation with stress and Young’s modulus (collinearity with the stress)], and runners presenting short fibers and greater PA (greater PCSA) and long in-series tendon (i.e., Achilles tendon) are better suited for economical C. The small observed power due to the low sample size limited the cause-effect impact of our stepwise multiple linear regression. Furthermore, improving the muscle architectural gear ratio can increase the muscle work production, generating a high efficiency, and probably decreasing C. Our results also showed large correlations between Achilles tendon’s morphology and mechanical properties (CSA, stress, and Young’s modulus) and triceps surae muscles’ architecture (GM PA, GM FL, SO PA, and SO FL) with C. In addition, our results showed that C at submaximal running speeds (12 and 16 km h^–1^) was negatively correlated with Achilles tendon CSA and positively correlated with tendon stress, which demonstrates a better force distribution per tendon area.

## Data Availability Statement

The original contributions presented in the study are publicly available. This data can be found here: (10.6084/m9.figshare.16766797).

## Ethics Statement

The studies involving human participants were reviewed and approved by Universidade Federal do Rio Grande do Sul. The patients/participants provided their written informed consent to participate in this study.

## Author Contributions

EM was responsible for the design of the study, data analysis and interpretation, drafting the manuscript, and revising it critically for important intellectual content. FL was responsible for the study conception, data acquisition, analysis and interpretation, supervision, and critical revision of the manuscript. ES was responsible for the design of the study, data acquisition, analysis and interpretation, supervision, and critical revision of the manuscript. FS was responsible for the data acquisition and critical revision of the manuscript. JF was responsible for the interpretation of the data and critical revision of the manuscript. JG was responsible for the data analysis and interpretation, supervision, and critical revision of the manuscript. MV provided the infrastructure for the study and was responsible for the interpretation of the data and critical revision of the manuscript. LP-T was responsible for the study design, providing infrastructure for the study, analyzing and interpreting the data, study supervision, and critical revision of the manuscript. All authors contributed to the final approval of the submitted version.

## Conflict of Interest

The authors declare that the research was conducted in the absence of any commercial or financial relationships that could be construed as a potential conflict of interest.

## Publisher’s Note

All claims expressed in this article are solely those of the authors and do not necessarily represent those of their affiliated organizations, or those of the publisher, the editors and the reviewers. Any product that may be evaluated in this article, or claim that may be made by its manufacturer, is not guaranteed or endorsed by the publisher.

## Abbreviations

ATP: Adenosine Triphosphate
CSA: Achilles tendon cross-sectional area
DXA: dual-energy X-ray absorptiometry
FL: fascicle length
GL: gastrocnemius lateralis
GM: gastrocnemius medialis
MRI: magnetic resonance imaging
V̇O_2MAX_: maximal oxygen uptake
MVIC: maximal voluntary isometric contractions
C: metabolic cost of running
MTU: muscle-tendon unit
MT: muscle thickness
MTJ: myotendinous junction
V̇O_2_: oxygen uptake
PA: pennation angle
PCSA: physiological cross-sectional area
RE: running economy
SO: soleus
TE: tendon excursion
TA: tibialis anterior
VIF: variance inflation factors.
